# Genome-wide *Mycobacterium tuberculosis* variation (GMTV) database: a new tool for integrating sequence variations and epidemiology

**DOI:** 10.1186/1471-2164-15-308

**Published:** 2014-04-25

**Authors:** Ekaterina N Chernyaeva, Marina V Shulgina, Mikhail S Rotkevich, Pavel V Dobrynin, Serguei A Simonov, Egor A Shitikov, Dmitry S Ischenko, Irina Y Karpova, Elena S Kostryukova, Elena N Ilina, Vadim M Govorun, Vyacheslav Y Zhuravlev, Olga A Manicheva, Peter K Yablonsky, Yulia D Isaeva, Elena Y Nosova, Igor V Mokrousov, Anna A Vyazovaya, Olga V Narvskaya, Alla L Lapidus, Stephen J O’Brien

**Affiliations:** 1 St. Petersburg State University, Theodosius Dobzhansky Center for Genome Bioinformatics, 41 Sredniy prospect, St. Petersburg, Russia; 2 St. Petersburg Institute of Phthisiopulmonology, 2-4 Ligovskiy prospect, St. Petersburg, Russia; 3 Research Institute of Physical-Chemical Medicine, 1a Malaya Pirogovskaya ul, Moscow, Russia; 4 Moscow Institute of Physics and Technology, 9 Institutskiy per., Dolgoprudny, Russia; 5 Moscow Scientific-Practical Center of Treatment of Tuberculosis of Moscow Healthcare, 10 Stromynka ul., Moscow, Russia; 6 St. Petersburg Pasteur Institute, 14 Mira ul., St. Petersburg, Russia; 7 St. Petersburg Academic University, 8/3 Khlopina ui., St. Petersburg, Russia

**Keywords:** *Mycobacterium tuberculosis*, Genome variations, Mutation, Genetic diversity, Whole genome sequencing, Database

## Abstract

**Background:**

Tuberculosis (TB) poses a worldwide threat due to advancing multidrug-resistant strains and deadly co-infections with Human immunodeficiency virus. Today large amounts of *Mycobacterium tuberculosis* whole genome sequencing data are being assessed broadly and yet there exists no comprehensive online resource that connects *M. tuberculosis* genome variants with geographic origin, with drug resistance or with clinical outcome.

**Description:**

Here we describe a broadly inclusive unifying Genome-wide Mycobacterium tuberculosis Variation (GMTV) database, (http://mtb.dobzhanskycenter.org) that catalogues genome variations of *M. tuberculosis* strains collected across Russia. GMTV contains a broad spectrum of data derived from different sources and related to *M. tuberculosis* molecular biology, epidemiology, TB clinical outcome, year and place of isolation, drug resistance profiles and displays the variants across the genome using a dedicated genome browser. GMTV database, which includes 1084 genomes and over 69,000 SNP or Indel variants, can be queried about *M. tuberculosis* genome variation and putative associations with drug resistance, geographical origin, and clinical stages and outcomes.

**Conclusions:**

Implementation of GMTV tracks the pattern of changes of *M. tuberculosis* strains in different geographical areas, facilitates disease gene discoveries associated with drug resistance or different clinical sequelae, and automates comparative genomic analyses among *M. tuberculosis* strains.

## Background

Tuberculosis (TB) remains an ongoing threat to worldwide public health, which in 2011 caused some 8.7 new cases and killed 1.4 million people, including 430,000 co-infected with Human immunodeficiency virus (HIV) [1]. The incidence of multidrug-resistant strains is rising in spite of increasing financial resources being released to stem the epidemic. With globalization, improvement of the health care and epidemic control systems in one country may not guarantee prevention of this airborne disease in others. Russia reported 180,000 TB cases and 20,000 TB deaths in 2011 and shows the highest incidence of new multidrug-resistant strains developed largely due to noncompliant drug regimens [1,2].

Molecular genetic studies of *Mycobacterium tuberculosis* strains using various genotyping technologies offer an approach to monitor strain dispersal and evolutionary adaptations, important to stem bacterial and disease spread. Genetic markers that track TB transmission include IS6110, polymorphic GC-reach repetitive sequences, direct repeat regions and mycobacterial interspersed repetitive units [3-7]. Recently, it was shown that bacterial whole genome sequencing (WGS) provides greater discriminative power [8-11]. WGS of multiple isolates may address a broad range of topics – from questions on the transmission of clinical strains to how *M. tuberculosis* evolves over long and short time scales. Rapid analysis of WGS data allows to detect bacterial genetic variants based on single nucleotide polymorphisms (SNPs) and insertion/deletions (Indels), including mutations associated with drug resistance or genetic lineage.

Increasingly large quantities of genome sequence data are becoming available from different types of *M. tuberculosis* studies [12-16]. Numerous *M. tuberculosis* WGS studies have been used for phylogenetic analyses and to identify genetic factors involved in TB drug resistance [15,17,18]. Several databases were developed to systematize and compare genomic data. TubercuList (http://tuberculist.epfl.ch) database provides gene-based information of *M. tuberculosis* H37Rv genome [19]. Tuberculosis Database (TBDB http://www.tbdb.org) is an integrated database providing access to TB genomic sequence data and resources from *Mycobacterium* species and *M. tuberculosis* strains, relevant to the discovery and development of TB drugs, vaccines and biomarkers. Currently TBDB contains information about 21 mycobacterial species whole genome sequences, nine of which belong to *M. tuberculosis* complex [20]. Mycobacterial Genome Divergence Database (MGDD), allows to find genetic differences between two strains or species of *M. tuberculosis* complex [21]. A web-based comprehensive information system Pathosystems Resource Integration Center (PATRIC, http://patricbrc.org) provides comparative analysis for genomes of different bacterial pathogens, one of which is *M. tuberculosis*[22]. To date PATRIC contains 201 mycobacterial isolates whole genome sequences, 68 of which are *M. tuberculosis* genomes.

Although these TB information databases have been established, there is no comprehensive online resource that brings together detailed information on *M. tuberculosis* genome variations associated with phylogeographic distribution, drug resistance and clinical outcome of TB. Here we describe and release a broadly inclusive unifying database – Genome-wide Mycobacterium tuberculosis Variation (GMTV) – that catalogues genome variations of Russian *M. tuberculosis* strains combined with available clinical data. GMTV helps to discover genomic variants of *M. tuberculosis* strains from different geographical areas and lists genetic markers associated with drug resistance and different clinical TB signs. GMTV allows association analysis between molecular variation and clinical consequences as well as facilitating epidemiological surveillance of TB and HIV/TB co-infection. Our hope is that the database will allow to find efficacious strategies to control TB infection and spread.

## Construction and content

### Database construction

GMTV database contains a broad spectrum of data derived from different sources and relates to *M. tuberculosis* molecular biology, epidemiology, TB clinical outcome, year and place of isolation, and drug resistance profiles. Access to GMTV database is distributed through web application with Python backend that connected to our MySQL database. Every record in the database is identified by the unique sample ID. Each sample ID corresponds to the set of SNPs and Indels and other information (e.g. medical, geographical and drug resistance data). The database includes information from following databases: NCBI, KEGG metabolic pathways [23,24] and TubercuList [19], the web interface provides access to the corresponding websites through hyperlinks. The GMTV genome browser is an essential tool for genome variations visualization that could be used as an analytical tool to compare nucleotide variations. The core of our MySQL database is manually curated and contain *M. tuberculosis* genome sequences assessed at Theodosius Dobzhansky Center for Genome Bioinformatics (St. Petersburg), Research Institute of Physical-Chemical Medicine (Moscow) and publicly available data of sequenced *M. tuberculosis* strains obtained in Russia.

*Mycobacterium tuberculosis* H37Rv reference genome (NC_000962.3) was used for SNP and Indel calling. For reference assisted assembly sequence reads were aligned on reference genome (H37Rv) using bowtie2 program [25] with standard parameters (bowtie2 -x H37Rv - p30 -U raw_reads.fq -S aligned_reads.sam). For SNP calling and VCF file processing a combination of samtools and vcftools was used [26,27].

A web genome browser, GMTVB, based on JBrowse platform [28,29] is an essential component of GMTV. GMTVB allows one to compare distinct regions or genes among *M. tuberculosis* strains, including an option to select a particular reference sequence, e.g. H37Rv. GMTVB implements an AJAX paradigm, which increases reaction time. Distinctive regions, genes, genomic features are displayed in tracks. Some tracks are permanent for the reference (e.g. genes, CDS, repeats, etc.). The interface allows one to add, delete and substitute tracks as well as to change reference sequences. GMTV clinical and genetic data combined with graphical tools incorporated in GMTVB makes the database an effective instrument for TB analysis (available at http://mtb.dobzhanskycenter.org).

### M. tuberculosis isolates and genome sequence

Whole genome sequences from 1084 *M. tuberculosis* isolates with various medical datasets from different regions of the Russian Federation comprise the present database. The database contains information on 73 isolates sequenced by our research group and 1011 publicly available genome sequences.

**Figure 1:**
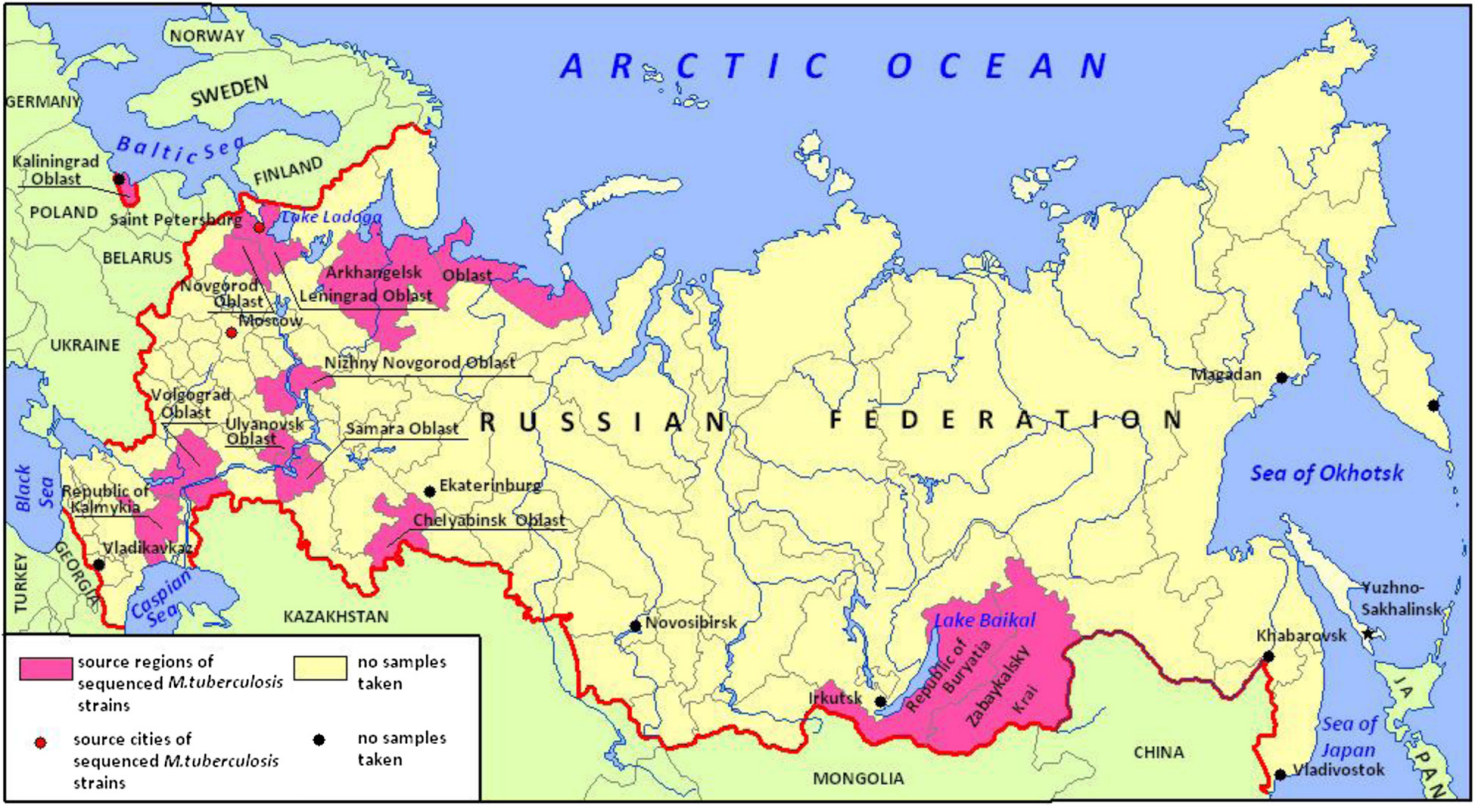
**Regions of the Russian Federation, from which *M. tuberculosis *isolates were obtained.** Source regions of *M. tuberculosis* strains genomes included into the database are highlighted with pink color.

Sequence data for 73 *M. tuberculosis* strains were provided by Theodosius Dobzhansky Center for Genome Bioinformatics (St. Petersburg State University) and Research Institute of Physical-Chemical Medicine. These *M. tuberculosis* strains were collected in St. Petersburg (n = 47), Leningrad Oblast (n = 7), Moscow (n = 7), Volgograd (n = 2), Kalmykiya (n = 2), Buryatya (n = 1), Arkhangelsk Oblast (n = 1), Chelyabinsk Oblast (n = 1), Kaliningrad Oblast (n = 1), Novgorod Oblast (n = 1), Nizhniy Novgorod Oblast (n = 1), Ulyanovsk Oblast (n = 1) and Zabaykalsky Krai (n = 1) (Figure 1). Bacterial isolates were provided by Saint-Petersburg Research Institute of Phthisiopulmonology (M. tuberculosis All-Russian Collection) and Moscow Scientific-Practical Center of Treatment of Tuberculosis of Moscow Healthcare. Genomic DNA of 73 *M. tuberculosis* strains was isolated using standard extraction method [4]. DNA samples were used for library preparation and sequenced using Illumina MiSeq Sequencing Platform and Roche 454 Life Sciences Genome Sequencer FLX following the manufacturer’s instructions. Sequencing data for *M. tuberculosis* sequenced genomes were deposited in the NCBI Sequence Read Archive [30] under accession numbers PRJNA218508 and PRJNA181180. The accuracy of these data stored at GMTV database is guaranteed by institutions that revealed the disease, performed microbiological tests and genomic data analysis (spoligotyping and WGS).

Whole genome sequence reads of other 1011 Russian *M. tuberculosis* isolates obtained in Samara region (Russia) were downloaded from the European Nucleotide Archive (http://www.ebi.ac.uk/ena/) submitted under accession no. ERP000192 [31,32]. The region of *M. tuberculosis* strains isolation and available drug susceptibility tests results provided by the authors were deposited to GMTV database.

**Figure 2:**
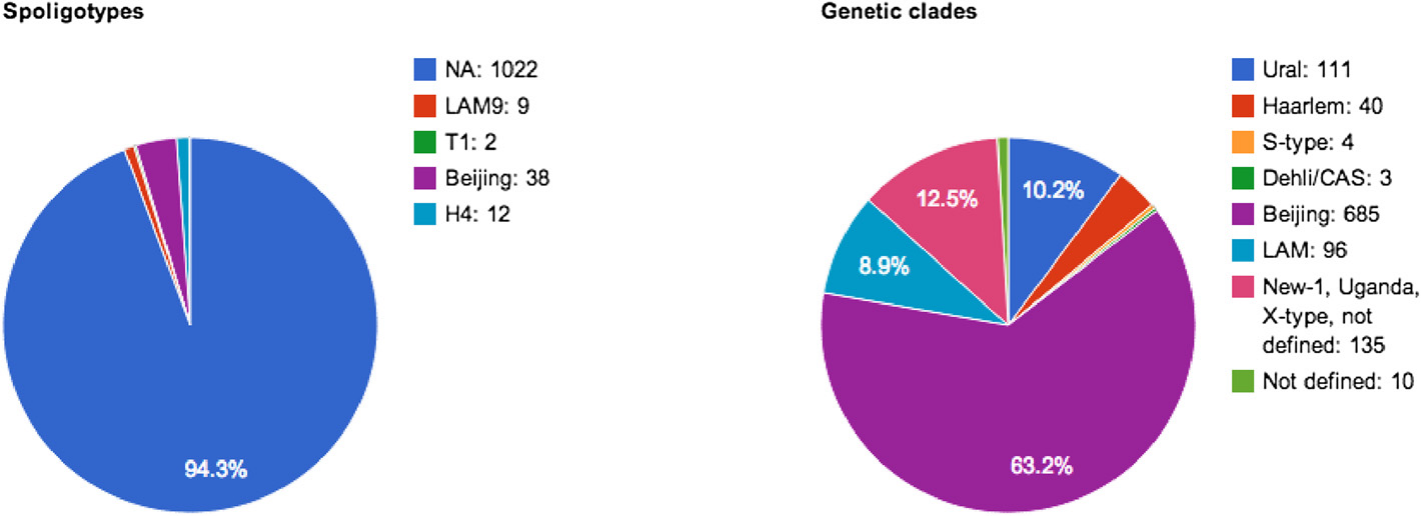
**Genotypes of *M. tuberculosis *isolates included into GMTV database.** Spoligotypes were identified using conventional spacer oligonucleotide typing technique and genetic clades were identified using SNP analysis.

Information about microbiological drug susceptibility tests is available for the majority of sequenced bacterial strains. Medical data were not available for all samples, however it will be updated as far as possible. To date GMTV database contains information on eleven isolates obtained from HIV-infected patients, 19 from HIV-negative, the rest of isolates were collected from people with unknown HIV status. Classical spoligotyping method was performed for 61 sequenced bacterial isolates [35]. Following spoligotype families were identified according to SpolDB4 database: Beijing (n = 38), H4 (n = 12), LAM9 (n = 9), T1 (n = 2) (Figure 2). Based on SNP analysis described earlier [16] we identified following genetic lineages among 1084 *M. tuberculosis* genomes: Beijing (n = 685), New/Uganda/X-type/not defined (n = 135), Ural (n = 111), LAM (n = 96), Haarlem (n = 40), S-type (n = 4), Dehli/CAS (n = 3), Not defined (n = 10) (Figure 2). Spoligotype International Type (SIT) number could be used to generate query for SNPs/Indels search. The clinical outcome of the TB was known for 30 isolates: 12 isolates were collected from patients with extrapulmonary TB, 15 from patients with pulmonary TB and 3 from patients with both pulmonary and extrapulmonary localizations. Four hundred eighty-seven isolates had proved multiple drug resistance (MDR) and 60 Extensive Drug Resistance (XDR). Limiting clinical data represented in the database without patient identifiers protects patients’ privacy.

## Utility and discussion

The current version of GMTV database has a web interface for the retrieval of genomic diversity, geography, drug resistance and clinical information. A derived genome variation table contains several types of information:

1. **Sample:** Sample ID, HIV status of the patient, patients’ gender, ear of strain isolation, spoligotype family name based on SpolDB4 [33], genetic clades based on SNP analysis [16], and geographical region.

2. **Genes:** Gene ID on NCBI database, gene name, locus tag, coordinates of gene start and end.

3. **Variations (SNPs and Indels):** SNP/Indel coordinates, Nucleic acid variations, Amino acid substitutions, Effect of the nucleic acid substitution (synonymous or nonsynonymous), various VCF file statistics, The SNP/Indels sections allows to download VCF file and easily get appropriate annotation of genome variations results. SNP, Indel or SNP/Indel options could be selected.

4. **Databases:** Information about protein function and protein functional category, according to TubercuList database [19] and Metabolic pathways, according to the KEGG database [23,24].

**Figure 3:**
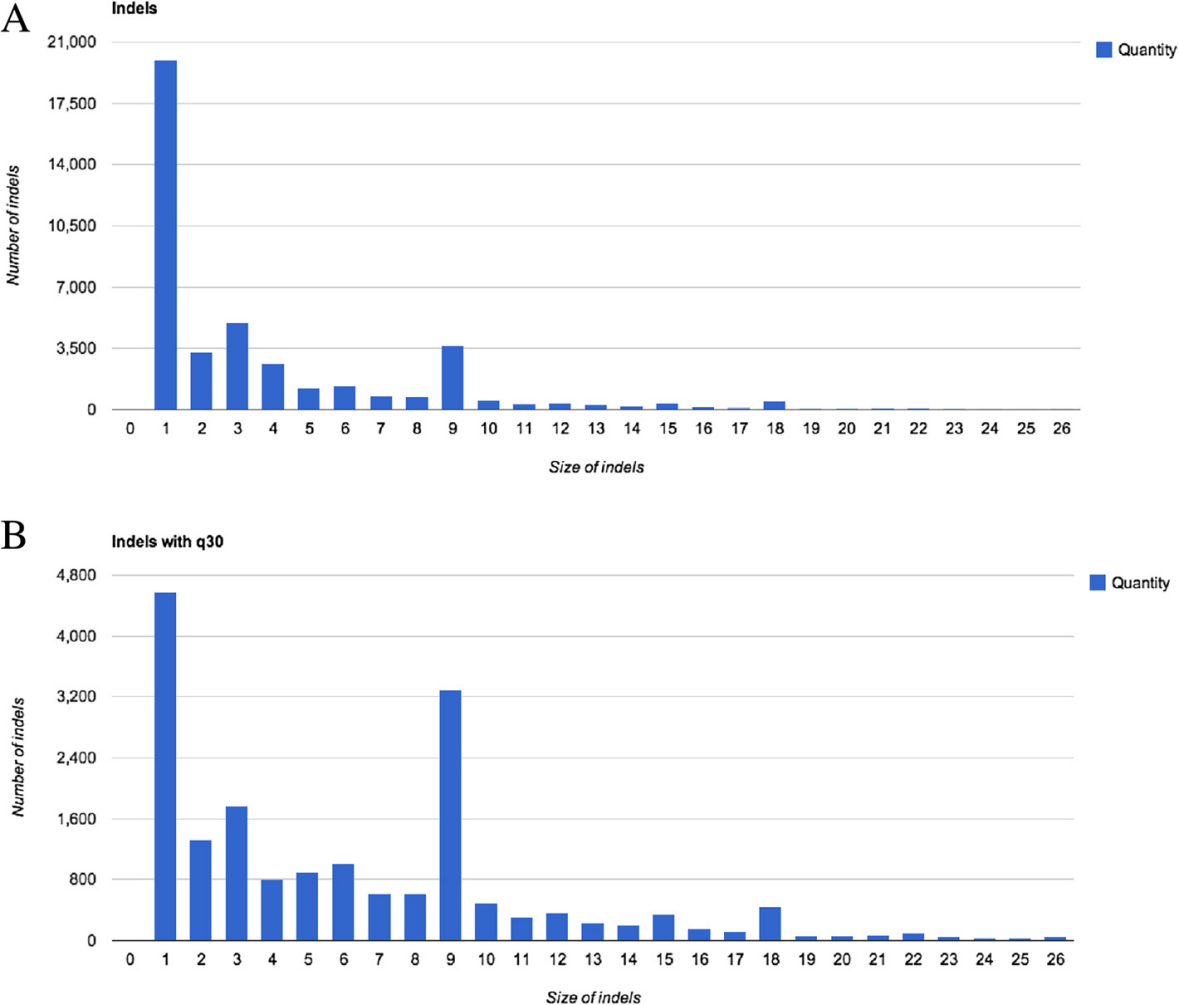
**Size distribution of Indels in *M. tuberculosis *genome. (A)** Indels distribution without Quality threshold, **(B)** Indels distribution with Quality threshold 30. One- and nine-nucleotide size Indels are the most common among *M. tuberculosis* isolates in GMTV database.

The input file for the database is a VCF file and a FASTQ file of the assembled genome, which can be downloaded from the website. Presently the GMTV database contains 1084 genomes and over 45,000 SNPs and 23,000 Indel variants across whole genomes with Quality (Q) score 30 threshold (Table 1). The Q score is one of the VCF file statistics associated with the probability of each substitution. The Q30 threshold means that the probability of incorrect base substitution is 1 in 1000. More than half of SNPs in coding regions (64%) are nonsynonymous. Analysis of Indels size, derived from VCF files, showed that one- and nine-nucleotide Indels are the most common, but other Indels are also widely spread (Figure 3).

**Table 1:** Genome variants (SNPs and Indels) in GMTV database filtered by Q30

**Genome variation**	**SNPs**	**Indels**
Overall quantity	45655	23975
In CDS	39808	18537
Nonsynonymous mutations in CDS	24124	-
Synonymous mutations in CDS	13392	-
Variations in STOP-codons in CDS	684	-
Frameshift mutations in CDS	-	10993

The GMTV web interface contains detailed information about genomic variations revealed from WGS data and provides for convenient analysis of genomic variation to facilitate searches of disease associations. Currently GMTV database allows:

• Review SNPs and Indels of *M. tuberculosis* isolates filtered by quality score and sequence coverage selected by the user;

• Identify functions and related metabolic pathways of genes where genome variations were identified using the links to KEGG and TubercuList databases;

• Compare SNPs between several isolates selected by drug resistance, clinical outcome, geographical distribution, genetic lineage and other characteristics. It is possible to select all, common or unique genome variations, synonymous and nonsynonymous mutations are highlighted;

• Annotate genome variations using integrated online-tool, download a table with annotated genome variations in CSV (Comma Separated Values) format for further research, and visualize results with the genome browser.

• Download VCF files with nucleotide genome variations, FQ files with reference-assisted assemblies of MTB genomes and FASTA files with sequences of selected genes.

**Figure 4:**
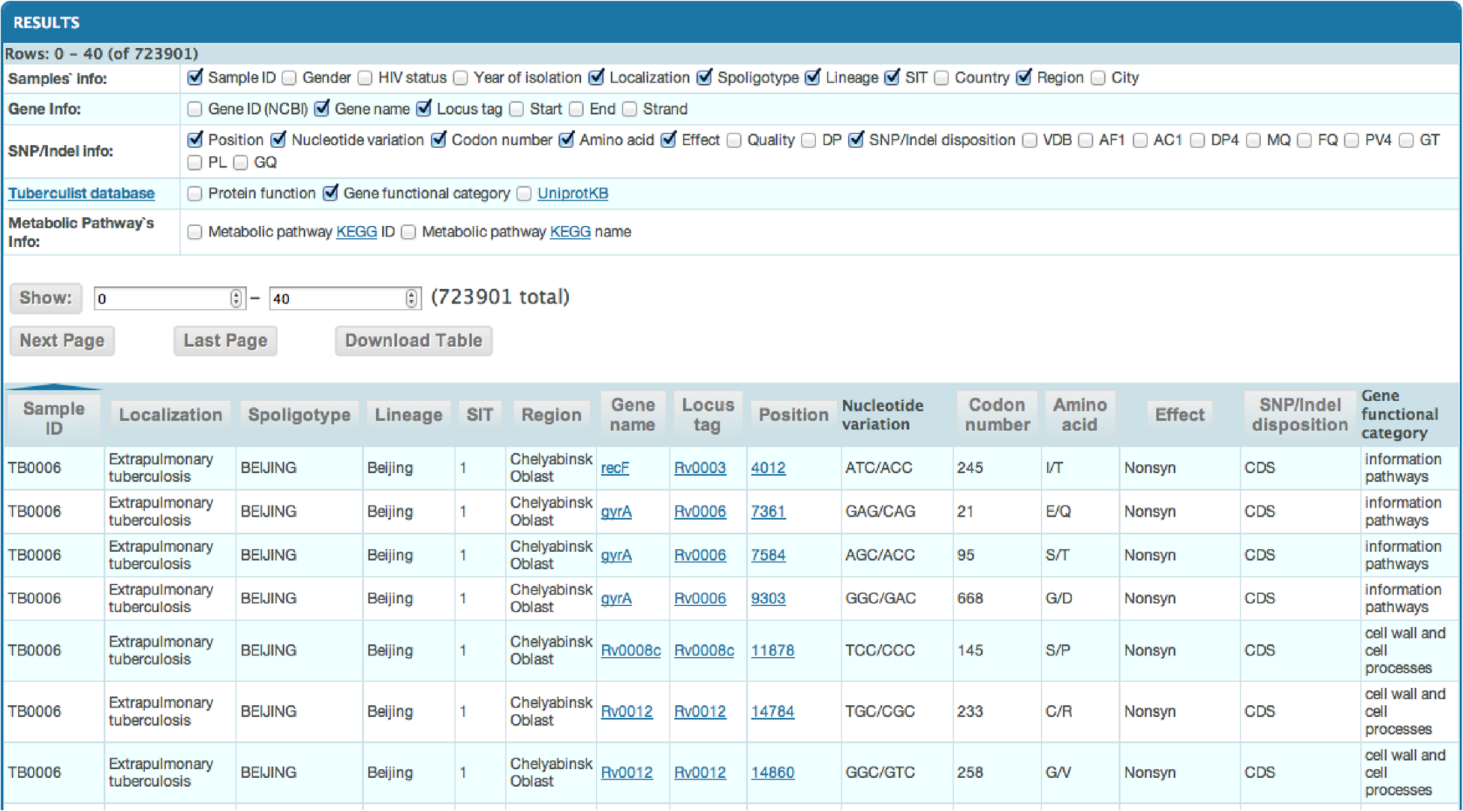
**An output of a query (SNP).** Query was based on SNPs in *M. tuberculosis* isolates with streptomycin and isoniazid resistance in coding regions with Quality threshold 30.

The work with the database starts from “Genome variations” page. To get required information the user can select a sample or some features (e.g. drug resistance, medical or genetic features) and click “Table” on the left bottom of the page. After generating the result, the user selects the type of information to be displayed in the top section of the page (Figure 4). There are five types of information which could be selected by the user: “sample info” provides medical, genetic and geographical information; “gene info” provides information about genes; “SNP/Indel info” provides statistics from VCF files; “Tuberculist database” provides information about genes functions; “Metabolic Pathways Info” provides available information from KEGG database. Information represented in the generated table could be filtered by sample ID, nucleotide position, gene name, geographical region etc. SNPs and Indels are analyzed in separate tables; the type of nucleotide variations could be selected in the top left section. It is possible to compare genome variations of bacterial isolates by downloading tables with mutations found in each group of genomes. The user may compare mutations in selected genes by listing several genes separated with “;” on the “Select genome region” section. This feature allows detecting mutations associated with drug resistance as well as finding compensatory mutations in other genes.

The “Download page” allows one to select some characteristics of *M. tuberculosis* isolates or ID of the interested isolates and to generate a table with information about genotype, geographic region and drug resistance. Each genome could be downloaded as a VCF file representing genome variations, or as an FQ file representing reference-assisted assembly of the genome. It is also possible to download FASTA files with a specific gene or genes, for this purpose the user have to select a special point “Gene” at the “Select genome region” section on the left bar region of the page and list one or several genes separated with ‘;’. This function is useful for comparative studies, for example, it allows analyzing selected protein-coding genes without intergenic regions.

The “Comparison samples” page is developed to compare single nucleotide genome variations. It is possible to browse variations in the whole genome sequence or gene-by-gene in selected *M. tuberculosis* isolates. For comparative analysis the user selects the sample ID in the left bar (the number is not limited) or to select interested features (medical, geographical, genotype or drug resistance). User may set Q score and coverage for SNPs. Generated table displays nucleotide variations and their position. It is possible to download the whole table or to browse mutations at the genome browser.

The combination of the database with a genome browser makes the GMTV an effective tool for interactive analysis and research. The browser illustrates a scalable map feature of the genome in tracks representing a site’s position, strand, value and supplement notes. Permanent tracks are preloaded into the browser (e.g. original DNA sequence, genes, repeats, etc. as well as defined SNPs, Indels) and listed on the left side of the screen. Tracks may be hidden or shown and there is an option to form tracks *ad hoc* based on SQL request. Such *ad hoc* tracks are created temporarily to provide opportunities to analyze the combination of data in visual form. Entire tracks can be downloaded in FASTA format. Scalable views of the genome elements inside the GMTVB let a user look at genome picture both with bird’s eye and as a detailed representation on the DNA level. GMTVB allows one to compare genomic features or to download selected feature for further analysis.

GMTV database is designed to assist in identification of genetic variants associated with drug resistance, clinical outcome or geographic distribution of the pathogen. It allows comparing nucleotide variations based on WGS data in different groups of *M. tuberculosis* isolates. Bacterial isolates could be divided into categories based on their geographic origin, drug resistance pattern, genetic clade or medical data. GMTV database functions allow using for phylogeographic, epidemiological and evolutionary studies.

GMTV is the first *M. tuberculosis* database to integrate clinical, epidemiological and microbiological description with genome variations based on whole genome sequencing data, a part of the large epidemiological database established at St. Petersburg Research Institute of Phthisiopulmonology. The development of a *M. tuberculosis* genome variations database will allow empirical exploring of influences of SNP and Indels around clinical outcomes. GMTV will facilitate the epidemiological surveillance of TB and HIV/TB co-infection and will help to develop effective strategies to control these infections in the population.

## Conclusions

GMTV allows association analysis between molecular variation and clinical consequences as well as facilitates epidemiological surveillance of TB and HIV/TB co-infection. Our hope is to inform efficacious strategies for TB control.

## Availability and requirements

The web server can be accessed at http://mtb.dobzhanskycenter.org.

## Competing interests

The authors declare no competing financial interests.

## Authors’ contributions

EC, PD, MR, SS developed the database construction. SS, MR performed web-based interface development, on-line annotation tool creation and JBrowse integration. PD, EC, ES and DI developed the pipeline and performed sequence reads analysis (*M. tuberculosis* annotations and reference-assisted genome assemblies). IK, EK and EC performed WGS of 73 *M. tuberculosis* strains. VZ, OM, IM, ON, AV, YI and EN provided *M. tuberculosis* samples, associated drug resistance, spoligotyping, epidemiological and clinical data on 73 *M. tuberculosis* isolates included in the GMTV. AL, EC, EI, VG, MS, PY and SJO designed the database. EC, ES, AL, MS and SJO wrote the manuscript. All authors read and approved the final manuscript.
